# Gender dysphoria in adolescence: examining the rapid-onset hypothesis

**DOI:** 10.1007/s40211-024-00500-8

**Published:** 2024-07-01

**Authors:** André Leonhardt, Martin Fuchs, Manuela Gander, Kathrin Sevecke

**Affiliations:** 1 Institute of Psychology, University of Innsbruck, Universitätsstraße 15, 6020 Innsbruck, Austria; 2 Department for Child and Adolescent Psychiatry, Medical University of Innsbruck, Innsbruck, Austria

**Keywords:** Gender dysphoria, Transgender, Gender identity development, Social contagion, Research controversy, Geschlechtsdysphorie, Transgender, Geschlechtsidentitätsentwicklung, Soziale Ansteckung, Forschungskontroverse

## Abstract

The sharp rise in the number of predominantly natal female adolescents experiencing gender dysphoria and seeking treatment in specialized clinics has sparked a contentious and polarized debate among both the scientific community and the public sphere. Few explanations have been offered for these recent developments. One proposal that has generated considerable attention is the notion of “rapid-onset” gender dysphoria, which is assumed to apply to a subset of adolescents and young adults. First introduced by Lisa Littman in a 2018 study of parental reports, it describes a subset of youth, primarily natal females, with no childhood indicators of gender dysphoria but with a sudden emergence of gender dysphoria symptoms during puberty or after its completion. For them, identifying as transgender is assumed to serve as a maladaptive coping mechanism for underlying mental health issues and is linked to social influences from peer groups and through social media. The purpose of this article is to analyze this theory and its associated hypotheses against the existing evidence base and to discuss its potential implications for future research and the advancement of treatment paradigms.

## Abstract (de)

Der starke Anstieg der Anzahl überwiegend weiblicher Jugendlicher, die an Geschlechtsdysphorie leiden und sich in spezialisierten Kliniken in Behandlung begeben, hat eine kontroverse und polarisierte wissenschaftliche wie auch gesellschaftliche Debatte entfacht. Bislang wurden nur wenige Erklärungen für diese jüngsten Entwicklungen vorgelegt. Ein Erklärungsansatz, der viel Aufmerksamkeit auf sich gezogen hat, ist die Idee einer „‚rapid-onset‘ gender dysphoria“, die eine Subgruppe von Jugendlichen und jungen Erwachsenen betreffen soll. Der Begriff wurde erstmals von Lisa Littman in einer Studie über Elternberichte aus dem Jahr 2018 eingeführt und beschreibt eine Subgruppe vorwiegend weiblicher Jugendlicher, die in der Kindheit keine Anzeichen von Geschlechtsdysphorie aufwiesen, bei denen die Symptomatik erst plötzlich während oder nach der Pubertät auftrat. In solchen Fällen wird vermutet, dass eine transgeschlechtliche Identifikation als maladadaptiver Bewältigungsmechanismus für zugrunde liegende psychische Probleme dient und mit sozialen Einflüssen von Peergroups und sozialen Medien in Verbindung steht. Ziel des vorliegenden Artikels ist es, diese Theorie und die damit verbundenen Hypothesen vor dem Hintergrund der vorhandenen Evidenz zu analysieren und mögliche Implikationen für die zukünftige Forschung und die Weiterentwicklung von Behandlungsparadigmen zu diskutieren.

## Adolescent gender dysphoria: current debates on the changing landscape

In recent years, specialized clinics have witnessed an unprecedented surge in adolescent patients seeking treatment for gender dysphoria [1–4]. Contrary to earlier trends where young natal boys displayed gender dysphoria from an early age, a marked shift has occurred, with significantly more natal female adolescents seeking treatment [3, 5–8]. Adolescents receiving clinical care for gender dysphoria are characterized by a considerable prevalence of co-occurring psychiatric disorders [9–12].

The evolving understanding of gender dysphoria is reflected in diagnostic developments. In the fifth edition of the Diagnostic and statistical manual of mental disorders (DSM-5) [13], the term “gender dysphoria” was introduced to replace the previous diagnosis of “gender identity disorder,” emphasizing that it is not gender identity itself but the distress caused by perceived gender incongruence that constitutes the condition. With the International Classification of Diseases 11 (ICD-11) [14], the former ICD-10 diagnosis “gender identity disorder” was removed and replaced with the new term “gender incongruence” in an effort to depathologize the condition. It was moved from Chapter F (“Mental and Behavioral Disorders”) to a separate chapter, “Conditions Related to Sexual Health,” outside the domain of mental disorders [15].

The demographic shifts and clinical developments we are witnessing among adolescents with gender dysphoria signal a paradigm shift and raise questions regarding its contributing factors. It necessitates a thorough re-evaluation of our theoretical models and treatment practices. However, this essential reassessment of theory and practice currently unfolds within a context marked by significant polarization, reflecting how the prospect of young people undergoing irreversible physical interventions carries the potential for evoking reactionary and emotionally charged responses. It might also mirror broader societal divisions on issues concerning sex and gender, sexuality and identity.

It is against this complex and contentious backdrop that the work of Lisa Littman emerged, offering an original lens through which to examine the recent trends in gender dysphoria among adolescents and caused substantial controversy. In her 2018 study [16], Littman introduced the notion of rapid-onset gender dysphoria (ROGD), arguing for its potential role in the uptick in youth with gender dysphoria, especially among natal females. For some individuals who develop gender dysphoria—Littman argues—preexisting mental health disorders, excessive consumption of social media content, and a clustering phenomenon among peers who collectively identify as trans might serve as contributing factors. Reactions to Littman’s findings are divided, ranging from those who see her findings as an important starting point for deeper investigation to critics who raise significant concerns about the study’s methodology as well as its potential to stigmatize and pathologize transgender identities.

By examining the origins, assumptions, and evidence surrounding the rapid-onset theory, this article aims to contribute to a nuanced understanding of adolescent gender dysphoria and the diverse approaches required navigating this complex field.

## The concept of rapid-onset gender dysphoria

The term “rapid-onset gender dysphoria” was initially coined by Lisa Littman in a 2018 publication [16], referring to a subgroup of adolescents identifying as trans and seeking clinical treatment for their gender dysphoria. In her study, Littman gathered data from 256 parents of adolescents experiencing gender dysphoria. These parents completed a survey including both multiple-choice and open-ended questions aimed at exploring shared experiences with their children (82.8% of whom where natal females), who suddenly showed signs of gender dysphoria during or after puberty without a prior history of issues related to their gender identity. Many parents noted that their children had previously been diagnosed with a mental health disorder or neurodevelopmental disability (e.g., autism) before the onset of gender dysphoria. A substantial portion of adolescents were described to be part of friend groups where a significant portion of the members simultaneously became transgender-identified, hinting at possible social and peer influences. Prior to identifying as trans, 63.5% of adolescents were reported by their parents to have significantly increased their social media and Internet use, where they were allegedly exposed to affirmative and persuasive narratives surrounding transgenderism.

In her study, Littman introduced the notion of a distinct adolescent subgroup experiencing rapid-onset gender dysphoria. This subgroup, primarily comprising natal females, is conceptualized as exhibiting a sudden onset of gender dysphoria, lacking any previous gender identity issues, but having preexisting psychological problems. Littman’s conclusions, drawn from parental reports, suggest that their children’s identification as transgender may be significantly influenced by an immersion in social media consumption and an ideologically homogeneous peer group with a shared belief that their trans identity was the cause of their mental health problems.

## Debates and methodological criticisms

The article was met with extensive criticism, particularly regarding its methodology [17–19]. Critics pointed out the reliance on parental reports collected through websites supposedly known to be critical of transgender identities, questioning the representativeness of the sample and the reliability of the observations reported. The pre-study communications to potential survey participants about the expected features of the subgroup [20] also drew criticism for potentially biasing the sample by attracting respondents with preconceived notions aligning with the study’s hypothesis. In response to the backlash, the journal conducted a post-publication reassessment of the article, which resulted in the publication of a correction of the article [21]. This correction included revisions to the Title, Abstract, Introduction, Discussion, and Conclusion sections, as well as an expansion of the Materials and Methods section to include new information and more details about the recruitment sites. The correction clarifies that recruitment information was distributed to sites with a variety of perspectives on gender dysphoria, including one that is perceived as pro-gender affirming. Furthermore, Littman emphasized the original purpose of her study, underscoring its hypothesis-generating rather than hypothesis-testing purpose. The fact that the article ultimately underwent two peer-review processes is a testament to the rigorous scrutiny Littman’s study was subjected to regarding its methodological soundness.

Despite the limitations and speculative nature of Littman’s findings, her theory of ROGD gained significant traction and sparked discussions that at times overlooked the scant evidence supporting it [22–24]. Even before the publication of Littman’s article, and thus before the results of the study were available, the phenomenon was discussed [25] and treated as established knowledge by prominent figures in the field such as Ray Blanchard and J. Michael Bailey [26]. This discrepancy between the significance of empirical findings from a single study and the far-reaching conclusions drawn from them is a valid point of criticism.

## A call for expanded inquiry

While the limitations of the study are clear, the use of parental surveys about their children is a longstanding and valid method within various fields of research for gathering preliminary data, informing hypotheses, and tracking behavioral trends. Therefore, the use of such surveys for hypothesis-generating studies should not be discouraged in clinical research, provided that one acknowledges their inherent limitations and potential for bias, especially in areas that involve very personal issues of identity. In clinical care, it is essential to complement parental data with insights directly from the adolescents and their clinicians. In the context of research on gender dysphoria, these additional perspectives are imperative for developing a more comprehensive understanding.

Littman [16] explicitly states that her study aimed to advance further investigation by generating hypotheses. Valid criticisms of the study and, at times, the handling of this relatively unsupported theory should not deter further research in this direction. Indeed, clinicians treating adolescents with gender dysphoria have noted observations in line with Littman’s description, prompting them to call for more research into this phenomenon [27, 28]. However, subsequent research on the ROGD hypothesis, such as a study by Diaz and Bailey [29], has also been heavily criticized and accused of serious scientific flaws. Following an open letter from transgender activist groups and researchers [30], the article by Diaz and Bailey was retracted. A recent study by Bauer et al. [31] tested the hypothesis of ROGD using clinical data from transgender adolescents. The study found no support for the hypothesis, suggesting that the phenomenon may not represent a distinct clinical condition. However, Littman [32] criticized the study, stating that Bauer and colleagues adopted a novel definition of ROGD by equating “recent gender knowledge” with “rapid onset” without relating the timing of the onset of gender dysphoria to puberty. This, she argues, undermines the study’s ability to provide meaningful information about ROGD.

Littman’s study introduces the hypothesis of a rapid-onset gender dysphoria subgroup, primarily consisting of natal females without prior gender dysphoria history, who begin exhibiting symptoms during or after puberty. She suggests that, for these individuals, gender dysphoria might act as a maladaptive coping strategy, with social influences—namely, social media and peer groups with a collective transgender identification—playing a significant role in the emergence of their transgender identity, akin to a form of social contagion. This leads to the consideration that a tailored approach in treatment may be necessary. The subsequent sections aim to critically examine and discuss the available evidence for these hypotheses.

## Rapid-onset gender dysphoria as a maladaptive coping mechanism

Maladaptive coping strategies refer to mechanisms or behaviors individuals employ to manage stressors or emotional discomfort that are ineffective or detrimental in the long term. Examples include excessive rumination or substance abuse [33–35]. In contrast to adaptive coping strategies, which address the root cause of distress and enhance resilience and problem-solving abilities, maladaptive strategies often provide only a temporary relief while potentially exacerbating the underlying problem. For the purposes of this discussion, it is worth noting that there is research indicating that natal females employ maladaptive coping strategies significantly more often than natal males [36, 37].

Evidence indicates a convergence of psychiatric disorders and gender dysphoria among some adolescents seeking treatment. The existing body of literature points to the significant prevalence of affective disorders (depression, anxiety) as well as self-harm and eating disorders [5, 12, 38, 39]. This confluence of conditions raises a critical question not only regarding the debate on ROGD but also the broader context of treating youth with gender dysphoria: Do preexisting psychiatric conditions act as a catalyst for gender dysphoria or are they rather the consequence of a preexisting, yet unidentified, gender dysphoria? On one side of the debate, the minority stress theory posits that the stigmatized social status faced by sexual minorities, in comparison with their heterosexual counterparts, culminates in heightened psychological distress [40]. Research suggests that co-occurring psychiatric disorders in youth with gender dysphoria may often be a consequence of minority stressors such as discrimination, harassment, and lack of social support [41–43]. Conversely, Littman’s research highlights parental reports suggesting that psychiatric issues in their children often predate the recognition of a transgender identity, with 62.5% of parents noting that their child had been diagnosed with at least one psychiatric condition before experiencing gender dysphoria. However, the reliability of parental perceptions in pinpointing the initial emergence of gender dysphoria remains contentious. Challenging the idea of a clear-cut onset of gender dysphoria in general, Preuss [44] argues for a more nuanced understanding, suggesting how gender dysphoria can be latent until the onset of puberty. Parents might miss earlier signs or not take them seriously, possibly even unconsciously communicating their displeasure to the child, causing the child to repress their feelings until puberty, when the distress emerges along with the sexual maturation of the body [45].

The notion of gender dysphoria as a maladaptive coping mechanism, as proposed by Littman, leaves open the question of its psychopathological scope, particularly in relation to disorders of identity development. Disorders of identity development involve significant difficulties in establishing a stable and coherent sense of self, often leading to psychological distress and impaired functioning [46]. Interestingly, research focusing on adolescents with gender dysphoria challenges the notion of a high prevalence of disorders of identity development. Karvonen and colleagues [47] found no evidence of increased problems with identity development among adolescents with gender dysphoria in comparison with clinical and non-clinical control groups. Similarly, a study by Haid-Stecher and colleagues [48] found no significant differences between patients in a transgender clinic and a clinical control group. However, they did observe indications of identity diffusion among some adolescents with gender dysphoria. Identity diffusion is characterized by fragile, contradictory descriptions of oneself and others and is a core feature of pathological personality organization [49]. This experience of a painful lack of coherence and continuity of the self is closely related to all severe personality pathologies [50]. Littman’s use of the term “maladaptive coping mechanism” needs to be clarified, especially given that gender dysphoria and a resulting but misguided transidentification would probably have to be classified as an identity disorder, and the available evidence does not really support its prevalence in the clinical population. Further research is needed to examine the relationship between identity diffusion and gender dysphoria in some adolescents to see whether they might correspond to the subset of adolescents hypothesized by Littman and to expand our understanding of these co-occurring conditions beyond the realm of coping strategies.

Criticism of Littman’s study often centers on its reliance on parental reports, thus disregarding the experiences of the adolescents themselves. To date, there has been no comprehensive empirical research merging the viewpoints of both the parents and their transgender-identifying children regarding the rapid-onset theory. However, experiences of “detransitioners”—individuals who have ceased or reversed their gender transition processes—can provide valuable insights [51, 52]. Their experiences shed light on potential connections between the initial onset of gender dysphoria and subsequent developmental trajectories, illustrating how different psychological, social, and medical factors may influence the emergence and progression of gender dysphoria. This emerging field of research reveals various reasons for detransitioning and provides insight into factors that may contribute to a rapid-onset gender dysphoria, such as social influences and psychopathology. These reasons range from the recognition that their gender dysphoria was intertwined with other factors (e.g., sexual orientation), insufficient information about the health risks associated with transition-related medical interventions, identifying past experiences of trauma or poor mental health as root causes of their dysphoria, or the sheer adversity of living in the desired gender role, even with medical support [53–55]. In particular, detransitioners’ claims that an underlying psychiatric disorder paved the way for them to “falsely” identify as trans are valuable in the context of a critical examination of Littman’s theory, although they appear to be in the minority among individuals (minors and adults) undergoing medical transition [56–58]. In one survey by Littman [53] with 100 participants, 40.6% of natal female adults and 32.3% of natal male adults who had pursued medical transition due to gender dysphoria and later detransitioned attributed their dysphoria to trauma, abuse, or other factors, with 40.6% of natal females (25.8% of natal males) arguing that their transgender identification was due to underlying mental health conditions. In a similar survey of adults [55], 70% of the 237 adult detransitioners included in the study (consisting of 92% natal females) acknowledged that their gender dysphoria had other causes, such as underlying mental health issues. Furthermore, 50% of the respondents indicated that transitioning did not help alleviate their gender dysphoria. Littman and colleagues [59] conducted another study in which they surveyed a sample of 78 adult participants (91% women) who had previously identified as transgender but had stopped doing so at least 6 months prior to the study. A total of 53% of the participants felt that their experience was consistent with rapid-onset gender dysphoria, indicating a sudden identification as transgender during or after puberty without significant childhood gender dysphoria. Many participants reflected on the lack of adequate assessment and consideration of transition alternatives, suggesting a need for more thorough assessment and discussion of the underlying causes of gender dysphoria. Lastly, a qualitative study by MacKinnon et al. [54] echoed these findings in a small sample of 28 adult detransitioners, with individuals attributing their gender dysphoria to other underlying issues, such as co-occurring psychiatric disorders and experiences of trauma as well as neurodevelopmental disorders (e.g., autism). The link to neurodevelopmental disorders corresponds with evidence of a disproportionate prevalence of autism spectrum disorders among youth and adults with gender dysphoria [60–62].

Given the limited research on detransition, which primarily relies on small and selective samples, any conclusions must be drawn with caution. Nevertheless, emerging studies underscore the complexity of gender dysphoria and suggest that, for some individuals, its origins may lie in psychiatric disorders, suggesting that transitioning, for them, might mirror something akin to the maladaptive coping mechanism Littman describes.

However, the proposition that trans-identification could act as a maladaptive coping mechanism for mental health disorders in certain individuals also raises numerous unresolved questions, particularly regarding the underlying causal relationship. The assertion that psychological vulnerability and susceptibility to trends and communities on social media or within peer groups fails to account for why only a very small minority of adolescents in such circumstances develop gender dysphoria. A significant number of adolescents are engaged with queer activist movements, and these engagements play an important role in identity formation for some of them, yet without ever leading them to question their own gender identity.

## Rapid-onset gender dysphoria and social contagion

Dishion and Tipsord [63] define “peer contagion” as a process of reciprocal influence among peers that includes behaviors and emotional exchanges that have the potential to negatively affect an individual’s development. Mechanisms include co-rumination, excessive reassurance seeking, and negative feedback seeking [64]. Research has consistently shown that co-rumination is particularly associated with an increase in mental health issues among natal females [64–66], linking it to conditions such as eating disorders [67, 68] and self-harm [69].

Warin [70] explores the intricate group dynamics among adolescents with anorexia, highlighting how the exclusion and marginalization of patients who adhere to therapeutic guidelines can inadvertently reinforce disorder-specific behaviors and beliefs. Against the background of evidence regarding the co-occurrence of gender dysphoria and eating disorders in some individuals [71, 72], Littman’s study raises a critical concern regarding possibly similar social influences and group dynamics among adolescents and young adults who identify as transgender. Littman’s study suggests that such dynamics could contribute to the development of gender dysphoria or a trans-identification in some individuals. Interestingly, a study by Vandenbussche [55] of adults who ceased their transitioning process revealed comparable interpersonal dynamics among the broader “queer community.” Some participants recounted difficulties in discussing the subject of detransitioning within community circles and describe feeling ostracized and losing their previous support system.

The impact of social media in the context of peer contagion has received more attention recently. The posting and sharing of graphic images of self-harm [73, 74] and content promoting anorexia [75] illustrate emerging digital dimensions of dynamic influencing processes. A growing body of research—largely based on correlational evidence [76]—points to the risks of excessive social media use, particularly among adolescent natal girls [77–80]. A longitudinal study by Leggett-James and Laursen [81] identified an association between social media use and a decrease in body image satisfaction among adolescents prone to peer influence, with significant effects emerging within a small span of about 13.5 weeks. Consistent with a recent meta-analysis [82], this study suggests that natal boys’ and natal girls’ body satisfaction is similarly influenced by social media use. These findings challenge the premise underlying ROGD that natal females are particularly susceptible to peer contagion processes via social media, and instead suggests a uniform susceptibility among adolescents. Haltigan, Pringsheim, and Rajkumar [83] underscore the idea of peer contagion among adolescents through social media platforms, highlighting the potential for self-diagnosis and the interplay between mental health issues and shared preoccupations with identity-related concerns. Interestingly, a recent study of a clinical sample of 165 adolescents with gender dysphoria found that the use of online support networks was associated with more internalizing problems [84].

The processes of peer contagion, particularly noted among adolescent natal females with psychiatric disorders, alongside the accumulating evidence of social media’s detrimental impacts on young people in general, give cause for consideration of the evidence emerging from Littman’s study. If it can be said that social contagion processes have been shown to play a role in certain disorders [67–69], and that social media can potentially exacerbate these processes [83], it is reasonable to assume that this may also be the case for some adolescents with gender dysphoria. This gives reason for closer study and, crucially, the direct involvement of adolescents themselves.

It should be noted that this examination of peer contagion processes among adolescents focuses on those with a DSM‑5 [13] diagnosis of gender dysphoria and deliberately avoids broader generalizations about transgender youth in general.

Finally, the potential of social media content to positively influence individual attempts to explore one’s identity and sexuality should not be overlooked. This exploration may be adaptive rather than maladaptive for some young people. Adolescents facing discrimination may find support in online communities, leading to the emergence of a self-understanding that may represent less the onset of a transgender identity through contagion than its realization.

## Rapid-onset gender dysphoria—implications

Zucker [85] identifies three critical questions raised by the ROGD theory: “First, is this really a new clinical phenomenon? Second, if it is, how do we understand it? Third, as a new clinical phenomenon, does it call for revisions to what are considered best practice therapeutics for adolescents with gender dysphoria?”

In our view, it is imperative to investigate the phenomena described in Littman’s research regarding a subgroup with distinct phenomenology. From our clinical experience in the medical care of affected children and adolescents, we observe at least a subset of patients with significant co-occurring psychopathology. It is not uncommon for these adolescents to describe an increase in gender dysphoria with the onset of puberty, often coinciding with the Covid-19 lockdowns and accompanied by an increase in social media use. In order to validate the theory and document the core clinical phenomenology, attempts must be made to replicate her observations using multiple sources of information (youth, parents, clinicians) and diverse methodology [85]. If forthcoming research substantiates the existence of this clinical phenomenon, the development of explanatory models will become crucial to enhance our clinical insight into the distinct needs of these patients.

A more nuanced exploration of the history of gender incongruence in early childhood may prove important for further investigation. Arnoldussen et al. [86] differentiated an early-onset group (average age around 12) and a late-onset group (average age just under 16) at the time of their first clinical presentation. The late-onset group demonstrated lower levels of recalled childhood gender incongruence, suggesting that, for some, gender dysphoria emerges in adolescence. This late-onset group warrants closer examination in future studies to discern potential patterns of psychodynamic differences from those with a history of (conscious) gender dysphoria since childhood. Further clinical investigation of adolescent-onset gender dysphoria and the distinct phenomenology may facilitate the subsequent differentiation of a potential ROGD subgroup. Future research should incorporate a more comprehensive diagnostic assessment of the patient population alongside a documentation of the circumstances leading up to the manifestation of the transgender identification. Interestingly, one aspect rarely considered in this context is the assessment of attachment patterns. Given that at least some adolescents may suffer from identity diffusion [48], which in turn is associated with insecure attachment patterns [50], research in this area would be useful.

Zucker [85] emphasizes that recognizing ROGD as a legitimate clinical phenomenon may necessitate a re-evaluation of the treatment guidelines for adolescents with gender dysphoria. It is essential to exercise caution when categorizing gender dysphoria into early-onset, late-onset, or rapid-onset subtypes. There is a risk that the legitimacy of an individual’s transgender identity might be questioned simply based on the categorical assignments by their clinician, potentially resulting in some being denied access to treatment in general. The significance of the initial manifestation of gender dysphoria (early vs. late) and its potential to serve as a predictor for likely developmental trajectories remains a topic of debate. In any case, the importance of psychotherapy in the treatment of adolescents with gender dysphoria, especially those with co-occurring psychiatric disorders, should be obvious [87]. In the clinical context, a thorough psychotherapeutic exploration provides a space for the individuals to examine the fantasies and motivations leading the individual to wish for hormonal or surgical treatment. Nonetheless, given the clear rise in the number of adolescents with gender dysphoria seeking treatment, alongside verified demographic shifts within this group, the emerging concept of an ROGD subgroup gives further reason to continually reassess existing treatment guidelines to ensure that they remain relevant and effective.

## Concluding remarks

This article aimed to examine the controversial theory of rapid-onset gender dysphoria (ROGD) in light of the existing evidence base. In summary, it becomes clear that neither prematurely adopting ROGD as a valid explanatory model nor its hasty condemnation as transphobic is an appropriate response. It is hard to deny that Littman’s research has made an important contribution to the discourse. It is now the task of the scientific community to take up this contribution and build on it with further research. We have to face the fact that ROGD may provide a convenient pathogenic explanatory model for those who are fundamentally opposed to medical transitioning of adolescents. In our opinion, however, the correct response to such possible tendencies is not to suppress research in this direction, but to strengthen it, so that evidence-based judgments of its validity are possible.

The complexity of gender dysphoria, particularly the proposed rapid-onset subtype, underscores the need for longitudinal, well-designed studies that can provide robust data over time. Such studies should aim to capture the multifaceted nature of gender dysphoria and its interplay with co-occurring psychopathology, social influences, and identity development.

Treatment in specialized clinics is inherently fraught with desires, anticipated disappointments, and anxieties [88]. Neither an attitude of generalized suspicion nor an uncritically permissive approach is adequate to meet the complex needs of adolescent patients with gender dysphoria. Given that interventions on young and healthy bodies often provoke strong emotional reactions from clinical staff, parents, and society at large [89], we must be wary of our own ideological predispositions. Ultimately, they dictate which explanatory models we consider more appropriate and which we reject.

From a perspective where clinical experience with adolescents with gender dysphoria and research in the field converge, we advocate further investigation of this phenomenon. Should Littman’s theory be substantiated, this conceptual framework can enhance our understanding and refine clinical treatment.
